# Treatment of Jehovah’s witness patients. Surgical and medico-legal considerations

**DOI:** 10.1186/1471-2318-11-S1-A62

**Published:** 2011-08-24

**Authors:** C Terranova, M Gruppo, F Mazzalai, R Lorenzetti, A Bruttocao

**Affiliations:** 1 Legal Medicine, Hospital - University of Padua, Italy; 2 Clinic of Geriatric Surgery, Hospital, University of Padua, Italy

## Background

Surgical treatment of Jehovah’s witnesses, even if elective, is a difficult situation for the surgeon from a clinical, deontological and ethical point of view. Any lesion or the death of the patient can result in civil and penal consequences for the surgeon and the anesthetist.

The aim of the present study was to present our experience regarding operative mortality and early clinical outcome in the surgical treatment of Jehovah’s witnesses compared to non-Jehovah's witness patients.

## Patients and methods

The research was structured as a retrospective cohort study. The total cohort analyzed was 80 subjects recruited from the Veneto Region, North-east Italy. Patients were divided into two groups:

Group 1, n=40, Jehovah's witness patients undergoing colorectal elective surgery.

Group 2, n=40, non-Jehovah's witness patients matched according to sex, age, type of surgery.

Informed consent was collected for each patient. Preoperative, intraoperative and postoperative conditions were analyzed. In particular, in the preoperative phase cardiovascular and hematologic conditions (hematocrit - Hk - and hemoglobin - Hb) were assessed; in the intraoperative and postoperative phases bleeding with severe anemization, length of stay in the intensive care unit and in our ward, re-admission rate, and mortality were considered.

## Results

All patients survived. No blood transfusions were administered to Jehovah’s witness patients. Severe complications and mortality rate were similar in the two groups. In particular, intra and postoperative bleeding, the need for intubation/resuscitation, and days of hospitalization were not significantly different (p> 0.05) in the two groups of patients. No malpractice claim was ruled.

## Conclusions

An accurate selection of patients, the minimization of perioperative blood loss, perioperative collection of autologous blood and meticulous surgical techniques could be responsible for the low rate of complications observed in both groups and the absence of claims of medical liability.
